# Paraneoplastic-Like Syndrome in a Breast Cancer Patient With a Negative Paraneoplastic Panel: A Case Report

**DOI:** 10.7759/cureus.40030

**Published:** 2023-06-06

**Authors:** Sydney Grubb, Aarti Joshi, Linette Hubbell

**Affiliations:** 1 Emergency Medicine, Alabama College of Osteopathic Medicine, Dothan, USA; 2 Obstetrics and Gynecology, Alabama College of Osteopathic Medicine, Dothan, USA; 3 Internal Medicine, Tallahassee Memorial Healthcare, Tallahassee, USA

**Keywords:** dyskinesia, paraneoplastic encephalitis, paraneoplastic syndromes, limbic encephalitis, paraneoplastic neurological syndromes, breast cancer

## Abstract

Breast cancer is a leading cause of cancer death in the United States, with an increasing incidence in recent years. Paraneoplastic syndromes are uncommon but increasingly recognized complications of many types of cancer, including breast cancer. Here, we describe a case of a patient presenting with confounding symptoms, who was diagnosed with breast cancer and believed to have a paraneoplastic syndrome despite a negative paraneoplastic panel. This case underscores the need for more standardized diagnostic modalities and prompt recognition and treatment of these rare but serious syndromes.

## Introduction

Breast cancer is the most common cancer in women (except for skin cancer) and the second leading cause of cancer deaths in women, second only to lung cancer. Although breast cancer can affect women of any age, the mean age of diagnosis is 62. Incidence rates have been increasing in recent years, but the overall death rate has been decreasing since the 1980s [1]. Multiple neurological and endocrine paraneoplastic syndromes have been reported to be associated with breast cancer. Treatment depends on the syndrome and etiology, and outcomes exhibit significant variability [2]. Here, we present a case of a healthy 72-year-old female patient with newly diagnosed breast cancer who developed dyskinesia and involuntary movements. Though these symptoms were in the setting of a negative paraneoplastic panel, she eventually improved with immunosuppressive therapy.

## Case presentation

**Figure 1:**
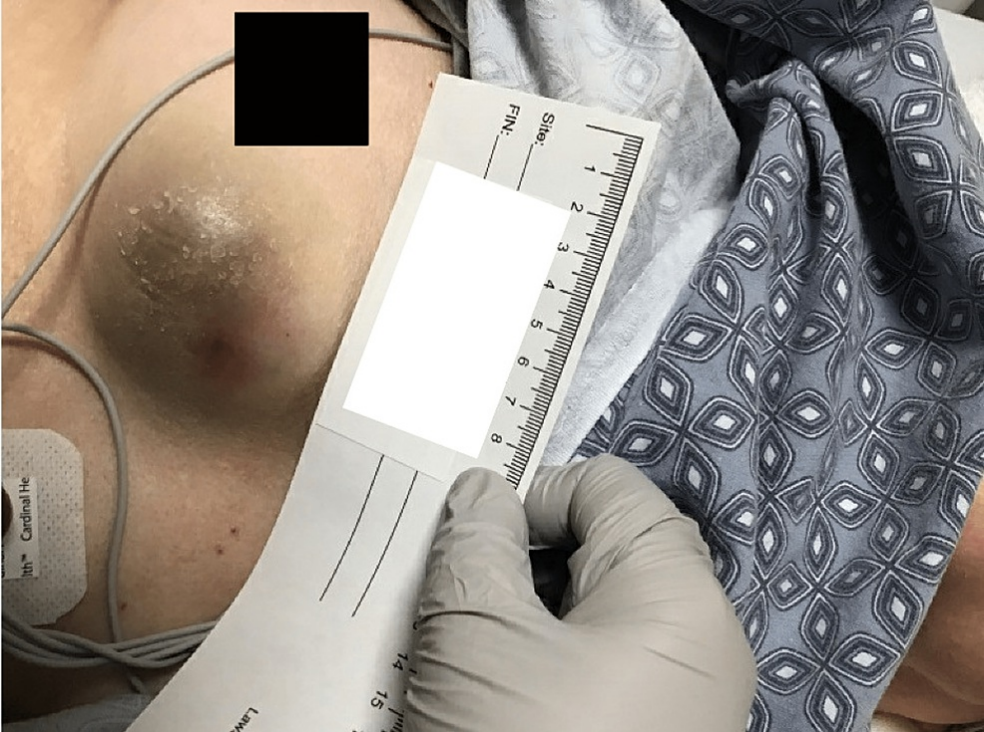
Image of the patient's breast upon admission

**Figure 2:**
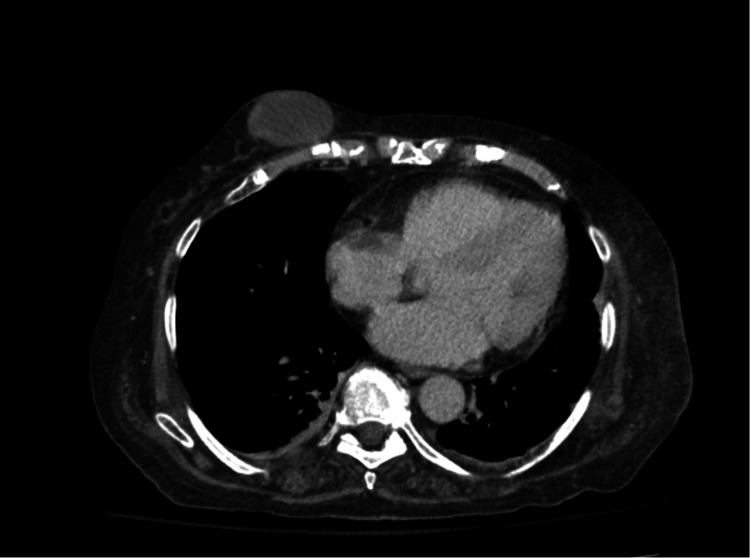
Chest CT of the patient at admission

A 72-year-old female presented to the emergency department (ED) with complaints of weakness and altered mental status. She reported that the weakness had been present for a week and had been progressively getting worse. She had a history of atrial fibrillation on diltiazem. On the day of admission, she began experiencing visual hallucinations, which prompted her visit. She had been seen in the ED a week prior for weakness and vertigo. She noted a lump on her breast, and a computed tomography (CT) scan showed a 3x5cm low-density subcutaneous mass on the right breast. The patient stated that the mass appeared eight months prior after she had a fall and had been progressively enlarging. In the ED, the patient was afebrile, and her vital signs were all within normal limits. She was oriented to self, location, and year but could not state the month or who the president was. She was noted to be restless and anxious, with diffuse weakness. The examination revealed a 3x5cm area of raised induration on the right breast with overlying bruising (Figure 1). Lab results showed an elevated sedimentation rate of 54. Electrolytes, white blood count, and hemoglobin were all within normal limits. A head CT scan showed no acute findings, and the electrocardiogram (EKG) showed normal sinus rhythm with no ST segment changes. Brain MRI was deferred at that time. A chest CT scan showed a 6.3x2.9x5.4cm lobulated mixed solid and cystic mass suggesting breast malignancy (Figure 2). Toxicology screen was deferred as the patient denied any current history of drug use. There was no history of insect or animal bites. There was no reported seizure activity, so EEG was deferred. There was no headache, fever, or nuchal rigidity, so lumbar puncture was not performed. She was admitted for further workup and management of weakness and the breast mass.

On the second day of admission, the patient became agitated but was able to be calmed by her family. Neurology performed an initial evaluation of the patient but as her confusion appeared improved, further management was deferred and observation was recommended. On the third day of admission, she developed violent involuntary movements in her bilateral lower extremities and bilateral upper extremities. The involuntary movements of the lower extremities were aggravated with active or passive movement. She additionally developed severe vertigo and was unable to ambulate. Neurology was called for a reassessment. A full neurological workup confirmed the presence of involuntary movements. Strength was ⅗ in the lower extremities and ⅘ in the upper extremities. Reflexes were normal in the upper extremities but were unable to be assessed in the lower extremities. She was given a single dose of 2mg intravenous lorazepam for dyskinesia. Neurology was concerned that her abnormal movements were related to a paraneoplastic syndrome, possibly anti-neuronal nuclear antibody-type 1 (anti-hu), so a paraneoplastic panel was ordered. A breast biopsy performed on the fifth day showed estrogen receptor (ER)/progesterone receptor (PR) positive, human epidermal growth factor 2 (HER2) negative invasive ductal carcinoma of the breast. Mammogram was not performed during this hospitalization. She was started on intravenous immunoglobulin (IVIG) therapy for presumed diagnosis of paraneoplastic syndrome. 

Additionally, she was started on anastrozole for breast cancer. She received five days of IVIG treatment, and her dyskinesia began improving on the second day and resolved on the fourth day of treatment. Interestingly, subsequent scans showed no metastasis of her cancer. The paraneoplastic panel was negative for amphiphysin antibodies, antiglial nuclear antibody 1 (AGNA-1), anti-Hu (ANNA-1), anti-ri (ANNA-2), anti-neuronal antibody 3 (ANNA-3), anti-CV2 (CRMP-5), neuronal (V-G) K+ channel antibodies, P/Q type calcium channel antibodies, anti-yo (PCA-1), Purkinje cell (PCA-2), and prostate cancer antigen (PCA-3). Following the five days of IVIG and the resolution of her dysarthria, the patient was discharged home. Oncology recommended surgical resection for her cancer, and she was discharged from the hospital with a plan to follow up as an outpatient for her breast cancer.

## Discussion

As the population of cancer patients continues to grow, the likelihood of encountering paraneoplastic syndromes is expected to rise. Paraneoplastic neurological syndromes (PNSs) are remote effects of cancer with immune-mediated pathogenesis. Breast cancer can occasionally result in paraneoplastic encephalomyelitis (PEM), which is a rare subtype of PNS. This condition can affect various areas of the central nervous system, including the hippocampus, lower brain stem, and spinal and dorsal root ganglia. Due to the diverse areas of the nervous system involved, the clinical presentation of PEM may have a broad range of neurological manifestations, such as limbic encephalitis (LE), brain stem syndromes, autonomic dysfunction, myelitis, chronic gastrointestinal pseudo-obstruction, cerebellar ataxia, and sensory polyneuropathy. Among these, LE, sensory polyneuropathy, and cerebellar ataxia are the most reported clinical presentations [3]. LE is the most frequent subtype of PEM and is characterized by an acute or subacute onset of symptoms such as confusion, seizures, and memory impairment [4].

The first time our patient presented to the hospital, she had neurologic symptoms characterized by weakness and vertigo. Several days later, she developed worsening weakness, visual hallucinations, altered mental status, and violent involuntary movements of the bilateral lower extremities and bilateral upper extremities. The subacute onset and overall findings are consistent with hypothesized LE.

Our patient meets the diagnostic criteria for a possible classical PNS, as outlined by the Paraneoplastic Neurological Syndrome Euronetwork. This classification pertains to neurological syndromes that are frequently associated with cancer. Notably, our patient presented with ER/PR positive, HER2 negative invasive ductal carcinoma in the right breast; however, the absence of onconeural antibodies (ONAs) highlights the diagnostic challenge in PNS cases. The criteria for definite PNS are defined as a classical syndrome and cancer that develops within five years of the diagnosis of the neurological disorder. In this setting, the presence of ONAs is not necessary for diagnosis [5]. While the presence of antibodies is not necessary to diagnose a PNS, it can create confusion and uncertainty surrounding the diagnosis, which can complicate the development of an effective treatment plan.

Thus, our case emphasizes the critical need for heightened vigilance in detecting PNSs, especially in cases where ONAs are not identified. Rapid identification and treatment of both the underlying breast cancer and overactive antibodies are vital for the best possible recovery outcome. Despite testing patient serum samples for ONAs using immunofluorescence, no antibodies were detected. Unfortunately, one-third of patients presenting with PNSs do not exhibit ONAs, making diagnosis and treatment challenging [5]. These findings indicate that further investigations may be necessary to develop more sensitive assays. Additionally, there is evidence that the Mayo Clinic Paraneoplastic Panel used in this case has a small percentage of false negatives, presenting further diagnostic challenges not only for this case but for other paraneoplastic syndromes [2]. The inability to detect ONAs in patients suspected of having PNS carries significant implications for their treatment, underscoring the need for more advanced and precise diagnostic techniques.

In terms of treatment, IVIG was found to be more effective in resolving our patient's neurological deficits compared to direct treatment of the tumor. Ambiguous diagnostic techniques can lead to improper management, which can delay treatment and result in irreversible neurological damage. However, there appears to be a consensus in the literature that addressing both the cancer and the overactive immune response in patients with suspected paraneoplastic syndrome is crucial for achieving the most favorable outcomes.

## Conclusions

Breast cancer remains a prominent contributor to cancer-related fatalities in the United States, with its incidence steadily increasing. Among the various complications linked to different cancer types, paraneoplastic syndromes have gained recognition, including their occurrence in breast cancer cases. Our patient meets the criteria for a potential paraneoplastic syndrome, exhibiting a clinical presentation that strongly aligns with limbic encephalitis. However, diagnosing this syndrome poses challenges due to the absence of ONAs in a significant number of patients. Even when these antibodies are present, the standard antibody panels may not consistently detect them. This case highlights the urgent necessity for comprehensive research and development to establish more standardized diagnostic techniques. Such advancements will empower health care professionals to achieve enhanced accuracy in their diagnoses and enable the initiation of timely treatment interventions.
